# Identity Document Presentation Attack Detection in Visible Light with Illumination-Controlled Scanner

**DOI:** 10.3390/jimaging12080396

**Published:** 2026-08-20

**Authors:** Lada Tolstenko, Alexey Popkov, Irina Kunina, Dmitry Polevoy, Sergey Usilin

**Affiliations:** 1 Federal Research Center “Computer Science and Control” of the Russian Academy of Sciences, 119333 Moscow, Russia; a.popkov@smartengines.com (A.P.); i.kunina@smartengines.com (I.K.); dvpsun@gmail.com (D.P.); usilin@smartengines.com (S.U.); 2 Smart Engines Service LLC, 117312 Moscow, Russia

**Keywords:** document presentation attack detection, scanner with controlled illumination, dynamic lighting, holograms, brightness normalization, flat-field correction, dark current correction

## Abstract

A reliable sign of the absence of a document presentation attack, when a print copy is presented instead of the original document, is the presence of such security features as OVDs (Optical Variable Devices), for example, holograms. To check the presence of holograms, it is sufficient to use the visible light and a series of document images captured with a varying angle of incidence and reflection of light. It can be achieved either by changing the position of the document or by changing the position of the illumination source. This work proposes a method for detecting holograms on identity documents using a scanner with controlled illumination. The method is based on obtaining a series of document images in various illumination modes and identifying features characteristic of holograms. To test the method, a dataset MIDV-Holo-Scan was collected by scanning physical documents used in the creation of the open dataset MIDV-Holo. It includes both documents with holograms, accepted in this work as originals, and documents without holograms, simulating an attack on document presentation. The proposed method for detecting attacks on document presentation achieves a quality of Accuracy = 100%, which surpasses the quality of the baseline method published with the MIDV-Holo dataset.

## 1. Introduction

In the modern world, identity document recognition systems [1,2] are widespread, and the demand for automating their authenticity verification is gradually growing. For example, airports already operate special kiosks [3] for automated passport control, equipped with a camera for capturing a person’s face and a scanner for acquiring a document image.

Thanks to the use of a scanner, controlled acquisition conditions are created [4], which, in turn, lead to higher quality performance of document analysis systems. For reliable document authenticity verification, multispectral scanners are used [5,6]. Document analysis in the visible light, supplemented by analysis results in the IR and UV spectra [7,8], allows distinguishing an original document from a fake one with high accuracy. However, there is an approach where document authenticity verification could be performed only in the visible light.

**Figure 1:**
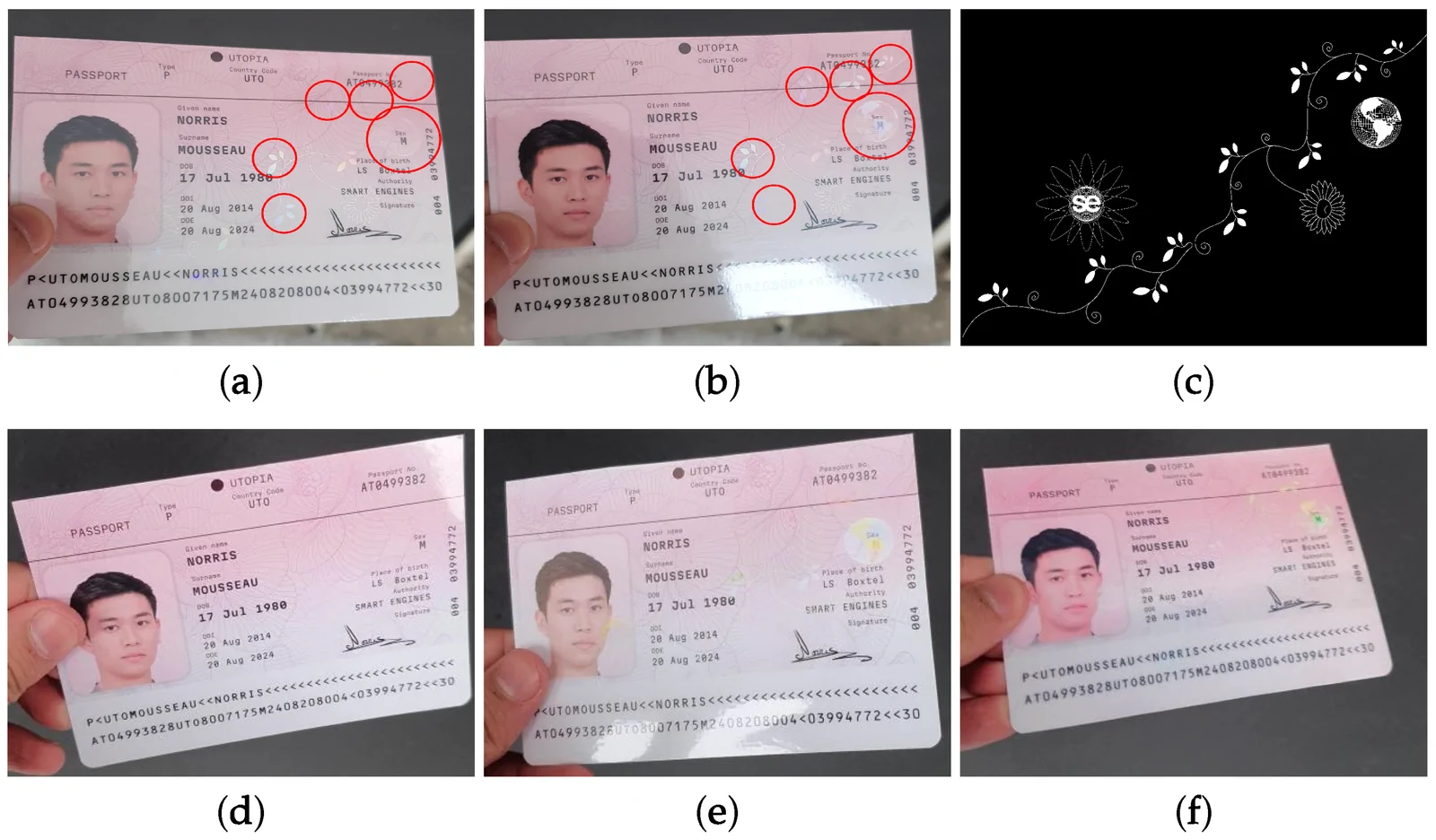
Examples of document images from MIDV-Holo [10]: (**a**,**b**) document with holographic security, captured from different angles (differing OVD areas are highlighted as red circles); (**c**) binary mask of the hologram used; (**d**–**f**) documents simulating attacks on document presentation: (**d**) document without a hologram, (**e**) document with a hologram image, (**f**) photocopy of a document with a hologram.

A reliable sign of the absence of an attack on document presentation (when a fake is presented instead of the original document) during image analysis is the presence of OVDs (Optical Variable Devices) [9] (Figure 1a–c). They are characterized by a change in color properties when the angle of incidence and reflection of light changes. Therefore, to detect an OVD, a corresponding sequence of images is required. The variability of the angle of incidence and reflection of light when using a scanner can be achieved by controlling the illumination and using lamps located at different positions under the scanning surface.

Within this publication, we propose a method involving a scanner with controlled illumination (Intek PS4 [5]) to detect attacks on document presentation based on the presence of OVDs. The method is tested on the MIDV-Holo-Scan dataset collected in this work, consisting of scans of 200 physical passport samples used in the creation of the MIDV-Holo dataset [10]. The tested documents include 50 samples with OVDs (Figure 1a,b), accepted in this publication as originals, as well as samples simulating attacks on document presentation (3 classes of 50 samples each) (Figure 1d–f). The obtained dataset is open (https://zenodo.org/records/20758652, accessed on 16 August 2026) and is published in this work.

Section 2 reviews the existing literature related to input devices, scanners, attacks on document presentation, OVD extraction algorithms, quality assessment methods, and datasets. Section 3 describes the published dataset of document images from a controlled scanner. Section 4 is devoted to the description of the proposed method for detecting attacks on document presentation. Section 5 presents the results of testing the baseline and proposed methods, as well as conclusions about the results of the conducted research.

## 2. Literature Review

One of the common types of attacks on document presentation is the use of a printed fake made on a home or office printer based on a digital image of the original document [11,12]. Unlike a simple photocopy, such fakes can reproduce the color scheme, graphic elements, and personal data of a document with high quality, which significantly complicates their visual detection. At the same time, reproducing OVDs, which change their appearance depending on the angle of incidence and reflection of light, remains a difficult task for an attacker. Therefore, one effective way to detect such attacks is to analyze the presence of OVDs on the presented document.

Optical Variable Devices [9] are special marks whose appearance depends on the position of the light source and the observer. These elements allow a person to quickly verify the authenticity of a document. A common type of OVD is a hologram [10,13], characterized by high saturation and variability of appearance when the angle of incidence and reflection of light changes. Hologram detection [10,13,14,15] and verification [16] are usually performed in a video stream using rule-based [10,17] and trainable methods [18].

The OVD detection task is typically solved on a sequence of frames from a mobile phone camera [10,13,14,15], where the viewing angle of the analyzed object changes from frame to frame. An important requirement for methods of detecting OVDs is pixel-wise alignment of the document across different images. In mobile capturing [19], conditions are uncontrolled [20], so achieving pixel-wise alignment is difficult. Fixing the document relative to the capturing device helps avoid this problem. There are a few works on using special devices for OVD detection [21,22], where the angle of incidence and reflection of light is varied by turning on lamps located at different positions. However, such devices are not as widespread as scanners.

In tasks of document analysis and authenticity verification, compact multispectral scanners are often used, which feature LED illumination around the perimeter of the working surface. In such scanners, instead of a scanning carriage used in conventional office (flatbed) scanners [23], a camera is used, which makes such scanners similar to planetary scanners [19] in terms of image acquisition. Thanks to this, the speed of image acquisition increases, which is important when working with a large volume of documents.

A trade-off between the devices considered is a compact scanner with controlled illumination.

For the purposes of this research, physical document fakes created for the MIDV-Holo dataset [10] are scanned. The MIDV-Holo dataset [10] is publicly available and consists of images of artificial documents obtained using a mobile phone camera. The dataset includes documents of two formats (passport and ID card), 200 samples each, with 10 unique templates for each format. MIDV-Holo [10] presents (Figure 1) both documents with OVDs (of the hologram type) and documents simulating presentation attacks: (1) laminated copy of a document without an OVD, (2) laminated copy of a document with a hologram, (3) document with a hologram image.

In the baseline method for detecting OVDs [10], the TPR (True Positive Rate) and FPR (False Positive Rate) functions are used to evaluate detection quality:

(1) $$TPR=\frac{TP}{TP+FN},\;FPR=\frac{FP}{FP+TN},$$

where *TP* is the number of correctly identified document samples with a presentation attack (samples without OVDs), *TN* is the number of correctly identified originals (samples with OVDs), *FP* is the number of originals falsely identified as a presentation attack, *FN* is the number of documents with a presentation attack falsely classified as originals.

In this work, scanned artificial passports from [10] are used, since there are no available datasets with pixel-wise aligned documents in images taken under different illumination modes. The quality assessment of the proposed OVD detection method is carried out in accordance with the reference publication [10].

## 3. MIDV-Holo-Scan Dataset

### 3.1. Controlled Scanner

**Figure 2:**
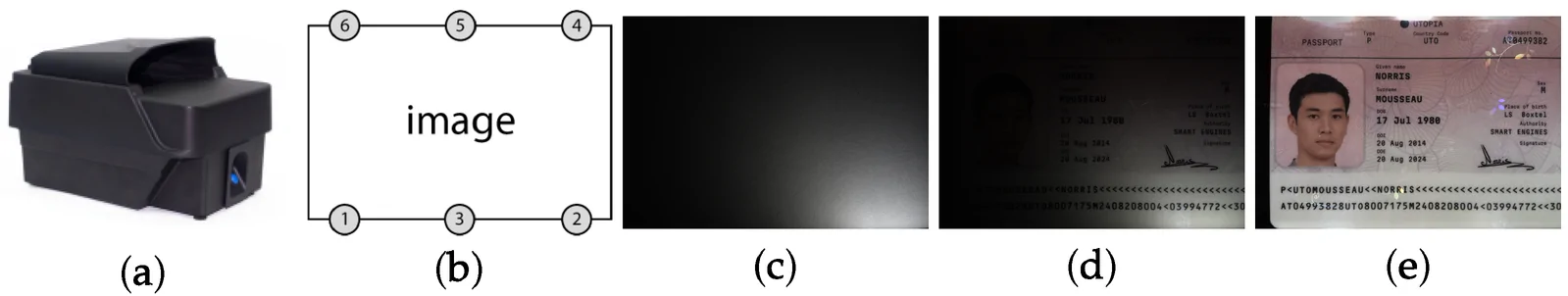
Scanner used: (**a**) external view of the scanner; (**b**) layout diagram of the lamps; example of illumination of a white sheet (**c**) and a document (**d**) when activating the same lamp; (**e**) example of an image obtained by the scanner’s standard software when activating all lamps.

For detecting OVDs in this work, a PS4 document scanner manufactured by Intek [5] is used (Figure 2a). The light source during scanning consists of six LED lamps, schematically presented in Figure 2b, whose relative brightness, as well as on/off state, can be controlled (Figure 2c–e).

### 3.2. Dataset Structure

To assess the quality of the proposed method, the MIDV-Holo-Scan dataset was created, containing scans of physical documents used in the creation of the MIDV-Holo dataset [10]. The set of scanned documents contained 200 passports, presented in 4 types (one type with OVD and 3 types without OVD simulating a presentation attack). Thus, the published dataset contains scans of 50 negative (with OVD) and 150 positive (without OVD) samples.

Let us call a scanning mode the set of active illumination lamps and their relative power values. This work considers modes in which exactly one lamp is active. To form the MIDV-Holo-Scan dataset, each document was scanned in all such illumination modes with variable (from 0 to 1 in steps of 0.1) relative power of the active lamp. Examples of images contained in the published dataset are provided in the next section. In addition to document scans, calibration images are added to the dataset, the details of which will be considered below. The obtained dataset is available at the link https://zenodo.org/records/20758652 (accessed on 16 August 2026).

## 4. Proposed Method

### 4.1. Main Steps of the Algorithm

The scanner’s standard software allows obtaining a document image in which OVDs are partially visible (Figure 2e). However, for reliable detection of OVDs, a single document image is insufficient (Section 2), and a series of document images with a varying angle of incidence and reflection of light is required.

**Figure 3:**
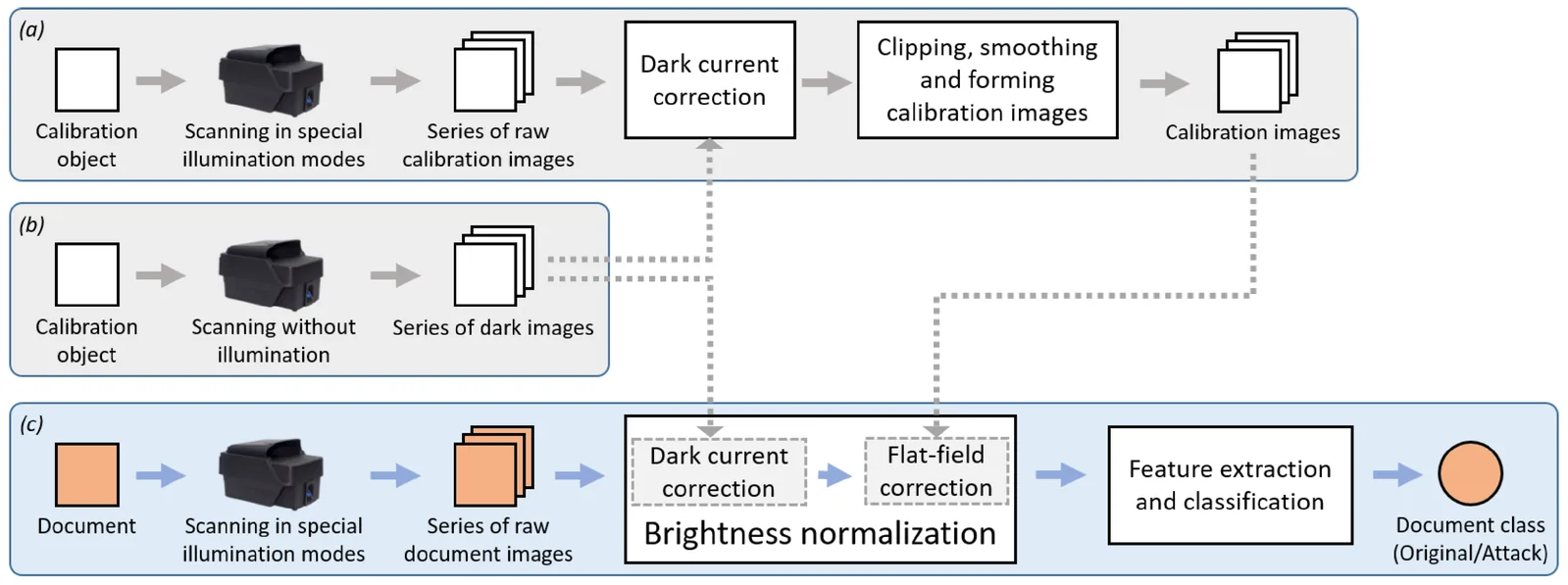
Scheme of the proposed algorithm: (**a**,**b**)—one-time preparation of a series of calibration and dark images, respectively; (**c**) the main pipeline of the algorithm using auxiliary images.

The proposed algorithm consists of four main steps (Figure 3c):

1. Acquiring a series of raw document images under different illumination modes;
2. Brightness normalization of the raw images in the series;
3. Extracting features indicative of the presence of OVDs;
4. Classification of the presented document into original/attack by the presence of an OVD.

For the second step, which is brightness normalization of the raw document images, auxiliary images of the calibration object are required. A series of 6 dark images scanned without illumination are used for dark current correction (Figure 3b). Calibration images (Figure 3a) are obtained by scanning in the same illumination modes used for document scanning, followed by special processing, and used for flat-field correction.

**Figure 4:**
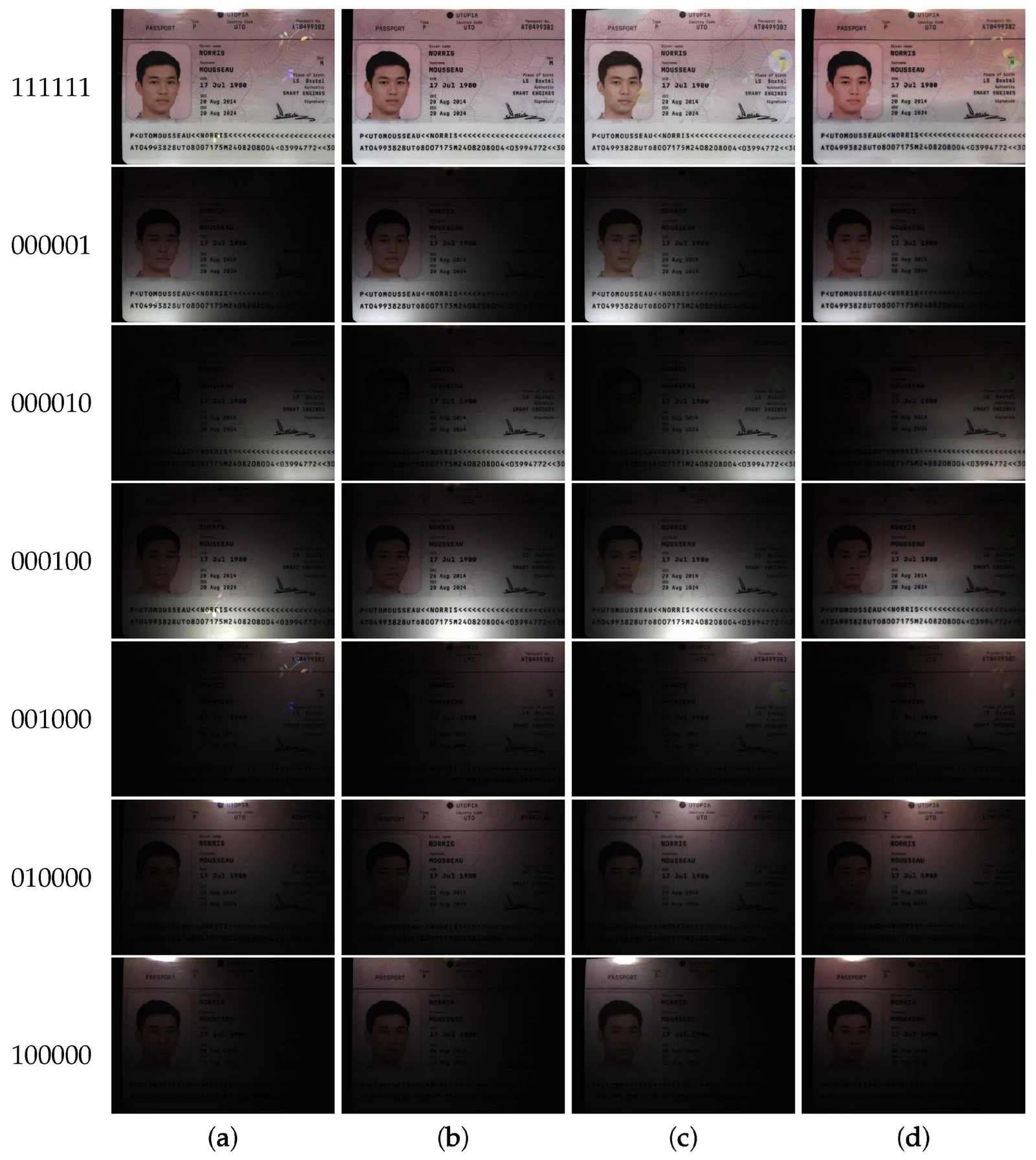
Examples of images by scanned document type, obtained in different illumination modes and by the scanner’s standard software: (**a**) document with hologram; (**b**) document without hologram; (**c**) document with hologram image; (**d**) photocopy of document with hologram.

Demonstration of the algorithm steps is carried out using documents from the published MIDV-Holo-Scan dataset (Figure 4), with holograms serving as the OVDs. The input images of the algorithm are normalized and have pixel brightness values in the range [0,1].

### 4.2. Illumination Operating Modes

**Figure 5:**
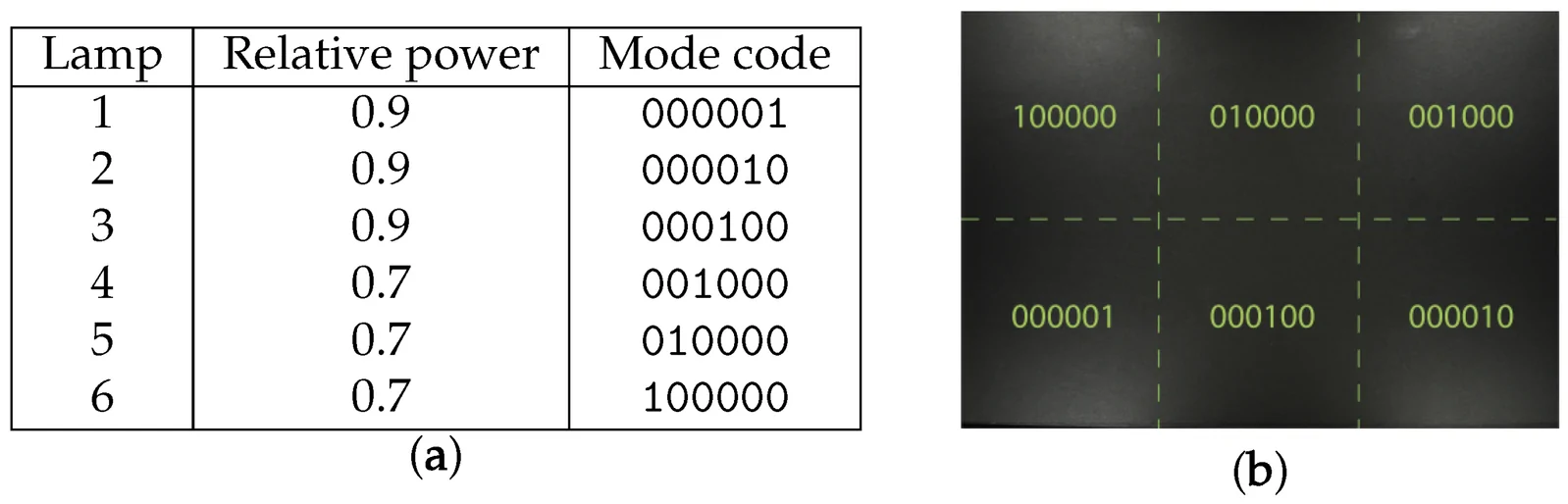
Illumination modes: (**a**) used relative lamp power; (**b**) layout diagram of areas $R_{k}$ of respective lamps.

In this work, 6 operating modes of scanner illumination are used (Figure 5a). Here, the mode is encoded by the binary representation of the active lamp number. The operating relative power values are chosen so as *to minimize areas with overexposed brightness values*. These modes are used when scanning documents, as well as at the stage of preparing calibration images.

### 4.3. Notations Used

Let us introduce the following notations. Let us call an image a set of pixels that are implemented as vectors in *m*-dimensional space $c=(c^{1},\ldots ,c^{m})$ depending on the Color Coordinate System (CCS) used. Then, the image is representable by a matrix C={c}, in which the color vectors c are organized in a certain order. Hereinafter, pixel-wise operations will be denoted using color vectors c, and reference to the entire image will be made through the same pixel notation *c*.

Images $C_{k}$ obtained from the scanner in mode $k=\bar{1,K}$, where *K* is the number of modes, are represented in the RGB CCS. Therefore, a pixel of such an image in mode *k* will be denoted as $c_{k}=\left(c_{k}^{R},c_{k}^{G},c_{k}^{B}\right)=\left(c_{k}^{1},c_{k}^{2},c_{k}^{3}\right)$.

### 4.4. Preparation of Calibration Images

**Figure 6:**
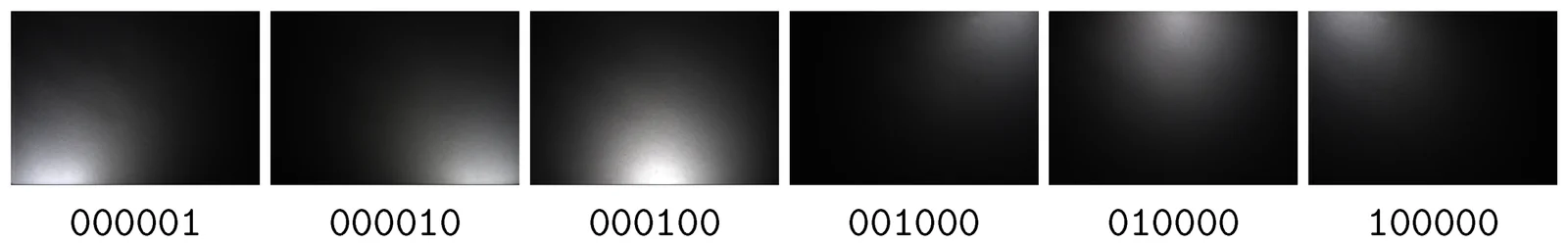
Calibration images for different illumination modes.

To prepare calibration images (Figure 3a), a calibration object is scanned in the modes considered in Section 4.2. A sheet of laminated white paper is used as the calibration object, emulating the surface of most documents, whose pages are either laminated or made of plastic. Prepared calibration images are demonstrated on Figure 6. Let us consider the obtaining of calibration images in detail.

#### 4.4.1. Dark Current Correction

At low illumination levels, dark current arises in the sensor, which leads to the false appearance of non-zero values. That is why a dark current correction operation is first applied to the series of raw calibration images. To estimate the dark current d, a set of 6 images $D_{k}$ taken with the illumination completely turned off is used (Figure 3b). Averaging over space and over the series is performed:   

(2) $$d=\underset{k}{M}\left[\underset{\left(x,y\right)}{M}\left[D_{k}\right]\right],$$

where M is the averaging operator. Dark current correction for image *C* is defined as

(3) $$c_{dark}=\max\left(0,c-d\right).$$

#### 4.4.2. Low Values Clipping

Then, low values are clipped according to the calib_threshold:

(4) $$u_{clip}=\max\left(\mathrm{calib}\text{\_}\mathrm{threshold},u\right).$$

#### 4.4.3. Smoothing

The next step is smoothing of the calibration images:

1. Downsampling by a factor of scale using bilinear interpolation;
2. Application of morphological operations Opening and Closing with a square kernel of kernel_size, followed by averaging the images obtained as a result of these operations;
3. Smoothing with a Gaussian kernel of size 2sigma+1;
4. Upscaling to the original size (by a factor of scale) using bicubic interpolation.

#### 4.4.4. Forming the Calibration Images

Finally, the calibration images $U_{k}$ are brought to the same average brightness level:

1. The average over the area $R_{k}$ (Figure 5b) of the calibration image *k* is calculated:
  (5) $$M_{k}=M\left[R_{k}\right];$$
2. The smoothed calibration image $U_{k}^{blur}$ is normalized for each mode *k* pixel-wise:
  (6) $$u_{k}=u_{k}^{blur}/N_{k},\;N_{k}=\max\left(\mathrm{target}\text{\_}\mathrm{brightness},M_{k}\right);$$
3. The calibration images $U_{k}$ are converted to single-channel $U_{k}=\left\{u_{k}\right\}$ by averaging across channels:
  (7) $$u_{k}=M\left[u_{k}\right].$$

### 4.5. Acquiring the Series of the Raw Document Images

Figure 4 shows examples of document images obtained using the scanner in different illumination modes: the standard scanner mode, conditionally designated as 111111, when all lamps are on, and those considered in Figure 5a from MIDV-Holo-Scan. OVDs in these images are quite noticeable, but the areas of the document opposite the light source remain dark with hardly distinguishable details. This is why normalization of raw images will be considered below by brightness level.

### 4.6. Brightness Normalization

**Figure 7:**
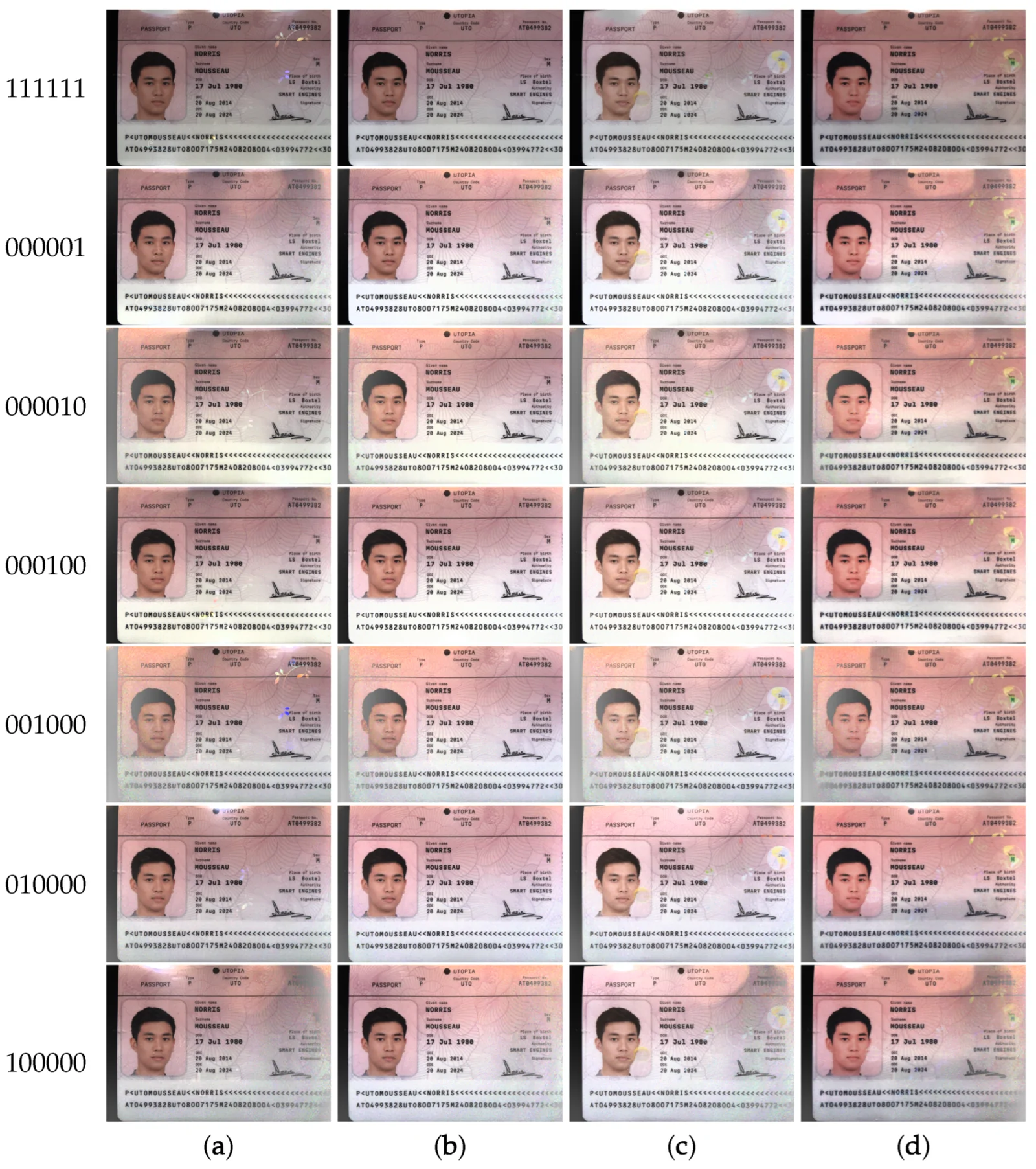
Series of normalized images by scanned document type: (**a**) document with hologram; (**b**) document without hologram; (**c**) document with hologram image; (**d**) photocopy of document with hologram.

Brightness normalization of the series of raw document images is performed in two stages (Figure 7): dark current correction (Section 4.4.1) and flat-field correction. The last one uses the prepared calibration images (Figure 6) to equalize the brightness of images $C_{k}$ for each mode:

(8) $$c_{k}^{calib}=\frac{c_{k}}{u_{k}}.$$

### 4.7. Extracting Features of OVDs

**Figure 8:**
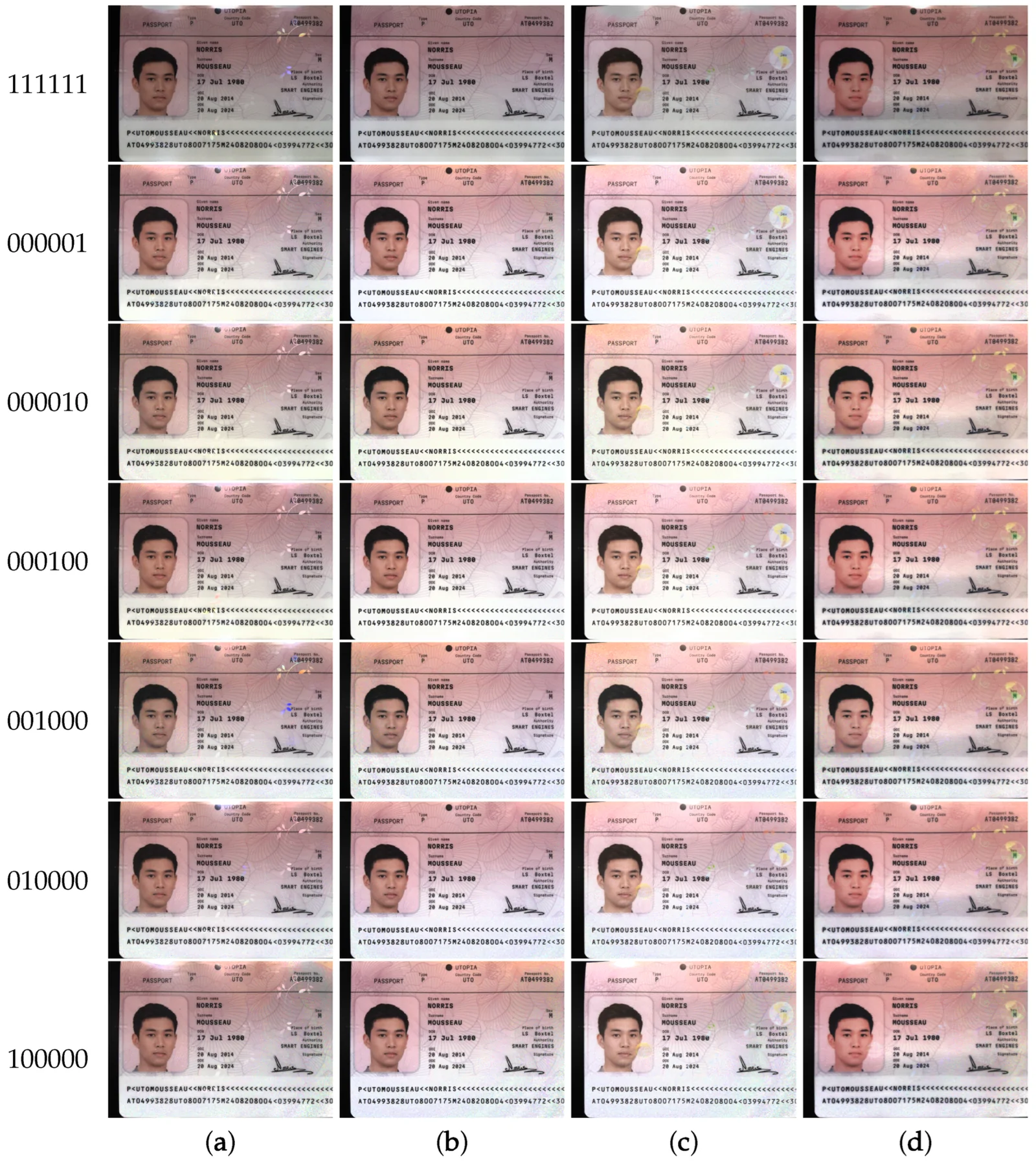
Series of images projected onto the plane of average brightness, by scanned document type: (**a**) document with hologram; (**b**) document without hologram; (**c**) document with hologram image; (**d**) photocopy of document with hologram.

To detect an OVD, the change in color between normalized images of the same series is calculated. The estimate of color change in a pixel is the standard deviation of the projections of color vectors onto the plane of average brightness (Figure 8), calculated over the set of normalized document images.

The projection of color vectors $c_{k}$ onto the plane of average brightness for each mode (Figure 8) is calculated by the formula:

(9) $$c_{k}^{\prime }=c_{k}\frac{M_{W}[∥c_{k}{∥}_{1}]}{∥c_{k}{∥}_{1}},\;W=\left\{U_{k}^{\mathrm{norm}\text{\_}\mathrm{param}}\right\},\;k=\bar{1,K},$$

where ${∥\cdot ∥}_{1}$ is the $\ell_{1}$ norm, and $M_{W}$ is the weighted average operator with weights *W*; exponentiation with parameter norm_param of calibration images $U_{k}$ is pixel-wise.

The calculation of the standard deviation of the color vectors projected onto the plane of average brightness is performed using the formula:

(10) $$s={\left(M_{W}\left[{\left(c_{k}^{\prime }-M_{W}\left[c_{k}\right]\right)}^{2}\right]\right)}^{1/2},\;k=\bar{1,K}.$$

**Figure 9:**
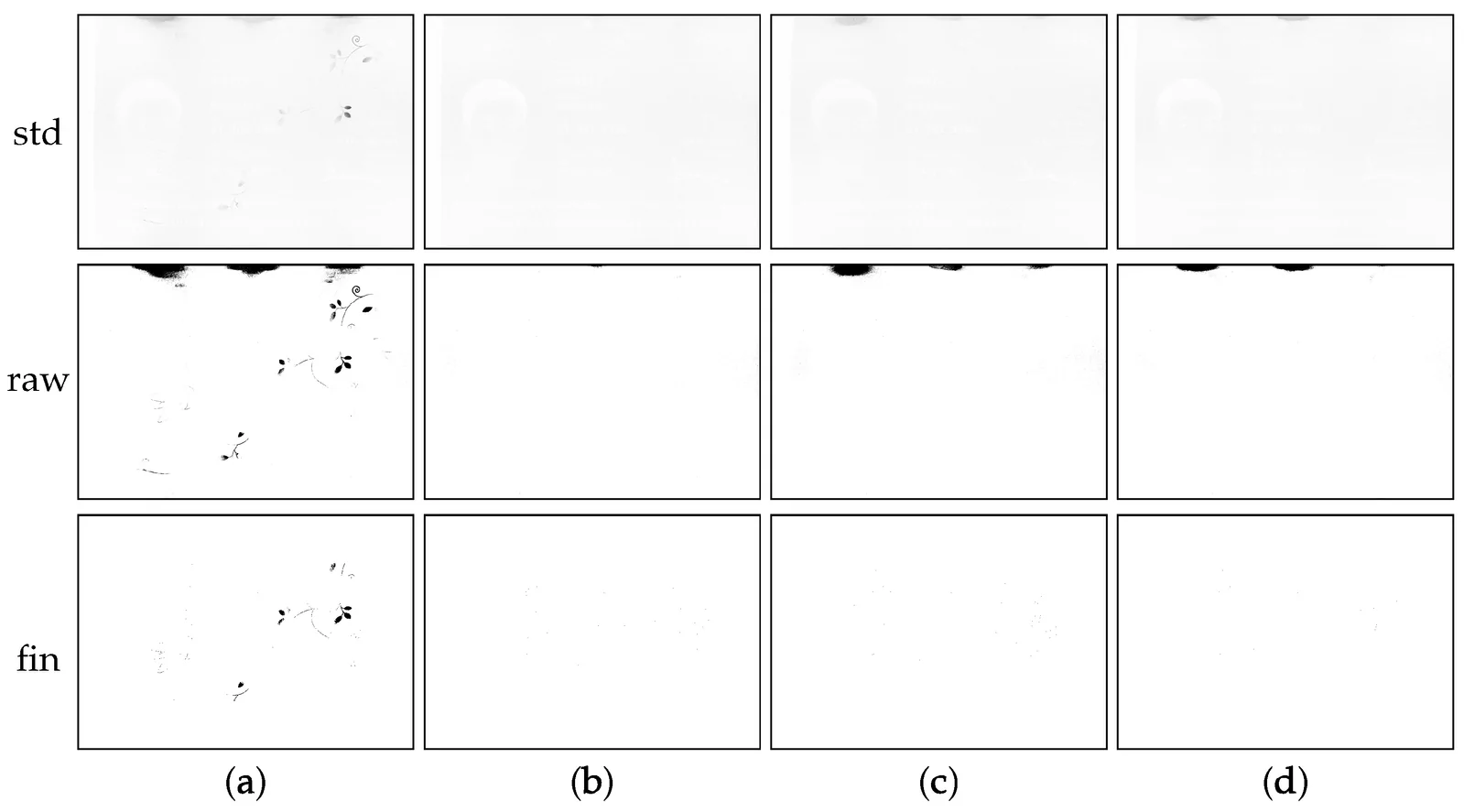
Inverted standard deviation map (std), raw OVDs mask (raw), and final OVDs mask (fin) by scanned document type: (**a**) document with hologram; (**b**) document without hologram; (**c**) document with hologram image; (**d**) photocopy of document with hologram.

From Figure 9 (std line), it can be seen that areas with OVDs have a higher standard deviation than areas without OVDs.

### 4.8. Making a Decision on the Presence of an OVD

The standard deviation map is reduced to the size doc_image_size. Let us construct a binary mask of OVDs *H* (see raw line of Figure 9), in which pixels have a value of 1 if the original pixel of the deviation map is above ovd_threshold, and 0 otherwise.

Let us introduce a rectangular area specified by the parameter ovd_roi in the format (left, top, width, height). We zero out the OVDs lying outside this area of the image *H* (see fin line of Figure 9).

Let us calculate the relative area of the found OVDs after zeroing and compare it with the specified threshold rel_area_threshold. If the relative area exceeds the set threshold, then we consider that the OVD is present on the given document. Thus, the document is classified as genuine (if OVD is present) or it is classified as a presentation attack (if OVD is absent).

## 5. Testing of OVD Detection Methods

### 5.1. Data for the Train and Test Phases

The collected MIDV-Holo-Scan dataset, a description of which is presented in Section 3, is divided into training (25%) and testing (75%) sets, preserving the document type distribution. Parameters are adjusted using the training set, which consists of scans of the first 10 documents of all four types (10 documents with OVDs, 30 documents without OVDs). The quality of the OVD detectors with the selected parameters is assessed using the remaining testing data.

### 5.2. Parameters of the Proposed Method

The resulting parameter values of the proposed method are shown in Table 1. The parameters ovd_threshold and rel_area_threshold were tuned on the training set during the maximization of the Accuracy function:

(11) $$Accuracy=\frac{TP+TN}{TP+TN+FP+FN}$$

with fixed values of the remaining parameters. The values of the remaining parameters were chosen based on the structure of the tested documents and the specifics of the images obtained from the scanner. In particular, one such parameter is ovd_roi. Its value is determined based on the characteristics of the documents being examined, specifically, the arrangement of OVD elements.

### 5.3. Baseline Method

As a baseline method, the hologram extraction algorithm published in [10] is used. This method was originally developed for analyzing frames obtained from a mobile phone camera. Despite this, the method [10] is applicable for working on images from MIDV-Holo-Scan, since the MIDV-Holo-Scan and MIDV-Holo [10] datasets contain images of the same documents on which the method [10] was tested.

**Figure 10:**
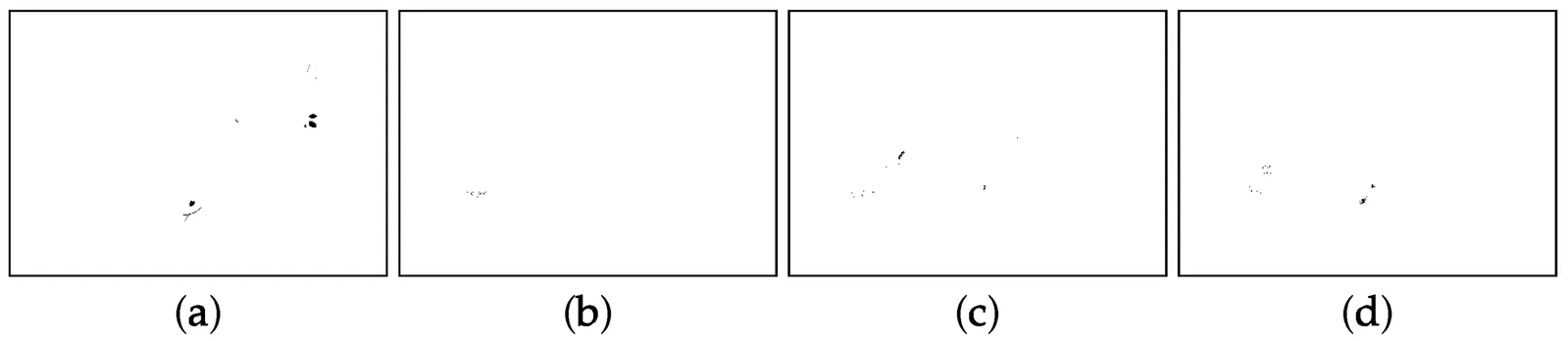
Inverted final mask of holograms generated by the baseline method, by scanned document type: (**a**) document with hologram; (**b**) document without hologram; (**c**) document with hologram image; (**d**) photocopy of document with hologram.

The baseline method was run on series of raw document images (Figure 4). The output of the algorithm is the mask of the found OVDs, based on the Hue and Saturation values [10]. To adapt this method to the characteristics of the images in question, the ovd_roi of the proposed method is used, as well as a tuned threshold ovd_threshold = 0.0035, according to which the input image is classified. The threshold is selected with use of the training set in order to maximize the Accuracy score, which is 75%. Examples of detected OVDs masks generated by the adapted method are shown in Figure 10.

### 5.4. Testing Results

Comparison of the quality of the baseline and proposed methods is performed on the testing part of the MIDV-Holo-Scan dataset. The performance quality of the baseline [10] and proposed methods is shown in Table 2.

The table shows that the proposed method outperforms the baseline method, which proves the effectiveness of the developed scheme for detecting document presentation attacks. The main problems of the baseline method are the low-quality response (see Table 3) to document types such as documents with a hologram image (32.5% of mistakes) and photocopy of a document with a hologram (55% of mistakes). A similar distribution of errors for the proposed method is not given due to the absence of errors (Accuracy = 100%).

## 6. Discussion

The proposed method detects presentation attacks from MIDV-Holo [10] with high accuracy. However, the number of attack types considered in this dataset is quite limited. Recently, the authors [18] published the MIDV-DynAttack dataset, consisting of documents with various distortions (e.g., a holographic sticker) that could be falsely accepted as OVDs. Thus, testing the algorithm on new types of attacks is required.

Furthermore, the proposed algorithm does not guarantee complete extraction of the OVD when constructing the mask. When comparing Figure 1c and Figure 9a (fin line), one can notice that the OVD is not fully extracted. This limits the application of this method in tasks such as verification of OVDs, in particular, holograms. Extraction of the OVD in full is another possible direction for future research.

## 7. Conclusions

In this work, we proposed a method for detecting document presentation attacks based on the presence of holograms using a scanner with controlled illumination in the visible light. For reliable detection of holograms, a series of document images obtained under different illumination modes is analyzed.

During the development of the method, the MIDV-Holo-Scan dataset was collected, consisting of 200 physical documents scanned in various illumination modes, corresponding to the documents from the previously published MIDV-Holo dataset [10]. A distinctive feature of the collected dataset is the pixel-wise alignment of the document across different illumination modes. This dataset is published with this work and can be useful not only in tasks related to documents but also in tasks of brightness normalization and image integration.

The quality assessment of the proposed method is carried out on the published dataset. The performance quality of the proposed method is TPR = 100% and FPR = 0%, which outperforms the baseline method [10]. However, the proposed method is not designed for counterfeit holographic elements, such as holographic stickers. It will respond to them as if they were genuine holograms. This limitation of the method can be circumvented by extracting the full hologram pattern and verifying its compliance with the expected one. Research into this issue is planned for future publications.

The high performance quality of the proposed method could be useful for application in different document authenticity verification systems. It highlights the relevance of developing new, cheaper devices, such as controlled scanners without additional spectra.

## Figures and Tables

**Table 1:** Algorithm parameters used during testing.

Parameter	Value
calib_threshold	0.05
scale	10
kernel_size	5
sigma	20
target_brightness	0.8
norm_param	2
ovd_threshold	0.06
doc_image_size	[3264, 2272]
ovd_roi	[482, 461, 2300, 1350]
rel_area_threshold	0.0015

**Table 2:** Obtained metric values on the testing set.

Detector	TPR, %	FPR, %	Accuracy, %
Baseline detector [10]	62.5	12.5	68.8
Proposed detector	100.0	0.0	100.0

**Table 3:** Error distribution of the baseline method by document type.

Type	FPR, %	FNR, %	Accuracy, %
Document with hologram	12.5	–	87.5
Document without hologram	–	25.0	75.0
Document with hologram image	–	32.5	67.5
Photocopy of document with hologram	–	55.0	45.0

## Data Availability

The original contributions presented in this study are included in the article. The MIDV-Holo-Scan dataset created in this study is publicly available at https://zenodo.org/records/20758652 (accessed on 16 August 2026). Further inquiries can be directed to the corresponding author.
